# Exploring the “gene–metabolite” network of ischemic stroke with blood stasis and toxin syndrome by integrated transcriptomics and metabolomics strategy

**DOI:** 10.1038/s41598-024-61633-y

**Published:** 2024-05-25

**Authors:** Yue Liu, Wenqiang Cui, Hongxi Liu, Mingjiang Yao, Wei Shen, Lina Miao, Jingjing Wei, Xiao Liang, Yunling Zhang

**Affiliations:** 1 Xiyuan Hospital, China Academy of Chinese Medical Sciences, Beijing, 100091 China; 2 Beijing Key Laboratory of Pharmacology of Chinese Materia Region, Institute of Basic Medical Sciences, Xiyuan Hospital of China Academy of Chinese Medical Sciences, Beijing, China; 3 Department of Neurology, Affiliated Hospital of Shandong University of Traditional Chinese Medicine, Jinan, China

**Keywords:** Ischemic stroke, Syndrome, Blood stasis and toxin, Traditional Chinese Medicine, Transcriptomics, Metabolomics, Diseases of the nervous system, Stroke

## Abstract

A research model combining a disease and syndrome can provide new ideas for the treatment of ischemic stroke. In the field of traditional Chinese medicine, blood stasis and toxin (BST) syndrome is considered an important syndrome seen in patients with ischemic stroke (IS). However, the biological basis of IS-BST syndrome is currently not well understood. Therefore, this study aimed to explore the biological mechanism of IS-BST syndrome. This study is divided into two parts: (1) establishment of an animal model of ischemic stroke disease and an animal model of BST syndrome in ischemic stroke; (2) use of omics methods to identify differentially expressed genes and metabolites in the models. We used middle cerebral artery occlusion (MCAO) surgery to establish the disease model, and utilized carrageenan combined with active dry yeast and MCAO surgery to construct the IS-BST syndrome model. Next, we used transcriptomics and metabolomics methods to explore the differential genes and metabolites in the disease model and IS-BST syndrome model. It is found that the IS-BST syndrome model exhibited more prominent characteristics of IS disease and syndrome features. Both the disease model and the IS-BST syndrome model share some common biological processes, such as thrombus formation, inflammatory response, purine metabolism, sphingolipid metabolism, and so on. Results of the “gene–metabolite” network revealed that the IS-BST syndrome model exhibited more pronounced features of complement-coagulation cascade reactions and amino acid metabolism disorders. Additionally, the “F2 (thrombin)–NMDAR/glutamate” pathway was coupled with the formation process of the blood stasis and toxin syndrome. This study reveals the intricate mechanism of IS-BST syndrome, offering a successful model for investigating the combination of disease and syndrome.

## Introduction

Stroke, an illness characterized by a high rate of morbidity, mortality, and disability, is the second leading cause of death globally^1^. Ischemic Stroke (IS) accounts for 87% of all stroke incidences and is the outcome of blood flow disruption caused by thrombotic and embolic events^2,3^. The recombinant tissue plasminogen activator (rt-PA) is currently the only approved medical therapy for IS. However, its clinical applicability is limited to only a small proportion of stroke patients by the narrow time window in which it can be administered^4,5^. As a result, the development of novel therapeutic drugs or combination therapies for IS treatment is imperative. With few therapeutic options available, patients and healthcare workers are increasingly embracing traditional Chinese medicine (TCM), which has a unique theoretical system characterized by a holistic concept and syndrome differentiation and treatment principles^6^. According to research, combining TCM and Western medication is effective in symptom relief, neurological healing, and enhancing IS patients’ Quality of Life (QoL)^7,8^.

The TCM concept is based on the fact that different stages of disease occurrence and development could present varying symptoms and signs. Syndrome (*ZHENG* in Chinese) comprises symptoms and signs that reflect the essence of a particular stage or type of disease. The various stages or types of syndromes intertwine and overlap, making up the entirety of the disease process. Developing a research model that integrates the “disease” concept in Western medicine with the “syndrome” concept in TCM theory is one of the future directions in Chinese integrative medicine. This approach aims to enhance our understanding of complex health conditions by harmonizing the perspectives of the two medical systems^9^. Blood stasis is an essential pathogenesis in TCM theory and clinical practice of IS, and the blood stasis syndrome (*Xueyu Zheng*) is the most common type of IS^10^. However, IS a dangerous condition that often progresses rapidly, and a single blood stasis theory may not comprehensively explain its complex pathogenic factors and processes^11^. The clinical IS manifestations caused by a sudden blood flow disruption are highly similar to those of diseases precipitated by ‘toxin’ in TCM. Illnesses caused by ‘toxin’ are often sudden and could even be fatal^12^. According to TCM, ‘toxins’ are formed by the accumulation and transformation of other pathogenic elements. Since blood stasis lasts for a long time, it could breed toxins. As a result, current IS practices stress the critical involvement of “blood stasis and toxin interaction” in its occurrence^13^. However, the biological mechanism underlying the Blood Stasis and Toxin (BST) syndrome remains unclear. Therefore, elucidating the biological basis of the IS-BST syndrome will undoubtedly promote advancements in the IS treatment methodology (Supplementary Information).

The Disease-Syndrome (DS) combination modeling is a crucial aspect of biomedical research^14^. According to the TCM basic theory, blood stasis could breed toxin, which in turn can consume body fluid, increasing blood viscosity and leading to blood stasis ultimately. Blood stasis and toxin accumulate in the body, leading to the occurrence of diseases. Modern medical research often explains this process as microcirculation disorder, abnormal hemorheology, enhanced platelet aggregation, inflammatory reactions, etc. Carrageenan (Ca) is considered to damage vascular endothelial cells and cause thrombosis, and is often used to prepare rodent thrombosis models^15,16^. Lipopolysaccharides (LPS) and active dry yeast (Yeast) acting on the body can induce the production of endogenous inflammatory factors and toxic substances, and are often used as tools in simulating the TCM concept of “toxin”. Our previous research utilized Ca to simulate the pathogenic factor of blood stasis, and used LPS and Yeast to simulate toxic pathogenic factors. We systematically compared BST models constructed using these three methods: simple Ca, Ca combined with LPS, and Ca combined with Yeast. We comprehensively evaluated syndrome characteristics, tail blood flow perfusion, whole blood viscosity, plasma viscosity, platelet aggregation rate, and plasma inflammatory factors, and ultimately found that the combination of Ca and Yeast models can present more stable BST syndrome characteristics. The BST model, established through the combination of Ca and Yeast, exhibited fever, a black tail phenomenon, reduced tail blood perfusion, elevated whole blood and plasma viscosity, increased platelet aggregation rate, and raised levels of the plasma inflammatory factor IL-6^17^. Based on these results, the present work aimed to establish a comprehensive animal model incorporating both the IS pathological characteristics and the BST syndrome characteristics. Specifically, we aim to create a fundamental tool for further studying the essence of IS and pharmacological mechanisms of Chinese herbal medicine. In other words, for a better understanding, it is important to conduct IS or syndrome-guided medication research on a mature DS combination model. However, diseases and syndromes are holistic concepts, and it is difficult to comprehensively describe the combination of diseases and syndromes using limited model evaluation indicators. As a solution to this drawback, omics technology has played an increasingly important role in life sciences in recent years, allowing the complexity of biological processes to be explained from multiple perspectives^18^. The application of “omics” in TCM research has attracted widespread attention, offering a technical platform for exploring the essence of the DS combination^19–21^.

**Figure 1:**
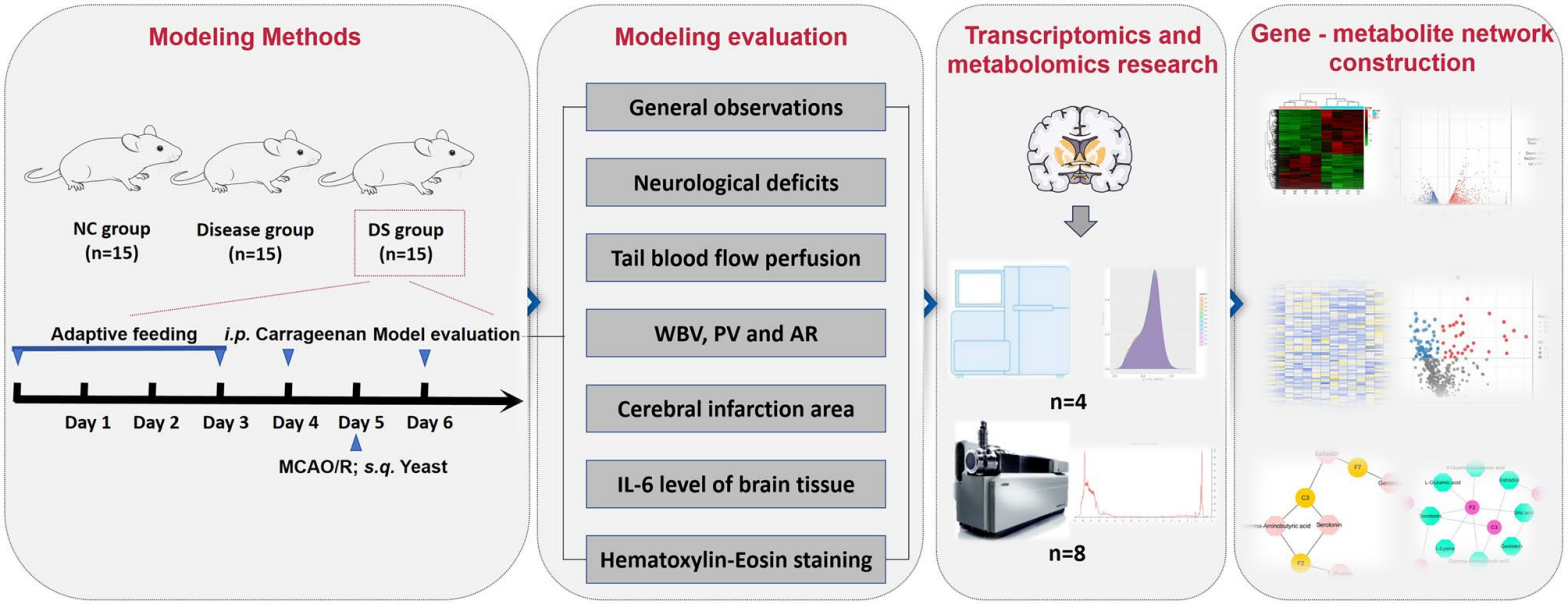
The flowchart of modeling methods and transcriptomics and metabolomics research.

Herein, we created a rat model incorporating both IS and the BST syndrome and designated it as the DS model. Transcriptomic and metabolomic approaches were used to investigate the biological basis of this model, yielding insights into the mechanisms underlying the IS-BST syndrome (Fig. 1). In addition to providing a scientific basis for TCM complexity, this study may discover new diagnostic biomarkers of the IS-BST syndrome, offering potential therapeutic targets for IS treatment.

## Materials and methods

### Experimental animals

Fourty-five male Specific Pathogen-Free (SPF) Sprague Dawley (SD) rats (weight = 230 ± 10 g) were purchased from Beijing Weitong Lihua Co., Ltd. [Beijing, China; Laboratory animal certificate number: SYXK (jing), 2018-0018]. These animals were housed in a controlled environment of 16 °C ± 2 °C and humidity of 55% ± 5% under a 12-h light/dark cycle and were allowed ad libitum access to food and water throughout the experiment. The Experimental Ethics Committee of Xiyuan Hospital ethically reviewed and approved this study’s research protocol.

### Reagents and materials

Carrageenan was supplied by Shanghai Macklin Biochemical Co., Ltd. (Shanghai, China, Batch No: C14408398). Active dry yeast was purchased from Angel Yeast Co., Ltd. (Yichang, China; Batch No: HY2009R). The rat Interleukin-6 (IL-6) Enzyme-Linked Immunosorbent Assay (ELISA) kit was acquired from Cohesion Biosciences. (UK; Batch No: CEK1619). Nylon suture for Middle Cerebral Artery Occlusion (MCAO) surgery was purchased from Hebei Tiannong Biotechnology Co., Ltd. (Shijiazhuang, China; Batch No: 20210630).

### Modeling and evaluation methods of disease and syndrome animal models

#### Modeling methods

After 3 days of adaptive feeding, the rats were randomly divided into three groups (n = 15): normal control group (NC group), the disease model group (Disease group) and the disease-syndrome model group (DS group).

The DS group was intraperitoneally injected with 10 mg·kg^−1^ of carrageenan on the first day of modeling. On the second day, the Disease and DS groups underwent MCAO surgery. Notably, the DS group was subcutaneously injected with 2 g·kg^−1^ of active dry yeast into the back immediately after inserting the nylon suture into the middle cerebral artery. On the other hand, the NC group was fed normally and received no treatment.

The modified Longa method was used to induce MCAO surgery^22^. The animals in the Disease and DS groups were weighed and anesthetized with pentobarbital sodium (40 mg·kg^−1^) after 12 h of fasting (water allowed). Subsequently, the anesthetized rats were fixed on the operating table, and the right Common Carotid Artery (CCA) and the right External Carotid Artery (ECA) were separated through a longitudinal incision in the middle of the neck and ligated near the cardiac end. A loose knot was then tied approximately 0.5 cm above the ligature. The Internal Carotid Artery (ICA) was clamped with an arterial clamp, and a small incision was made between the sliding and dead nodes of the CCA. The ICA artery clamp was tied off once the nylon suture was inserted and made contact with the CCA bifurcation. The suture entered the ICA from the CCA up until it reached the initial area of the anterior cerebral artery. The insertion was stopped when a small amount of resistance was felt from the incision, at approximately 18 mm. After 1.5 h of ischemia, the suture was removed from the ICA to the CCA, followed by 22.5 h of reperfusion.

#### Model evaluation indicators and methods

Model evaluation was performed as follows:

1. General observations: Mental status, diet, movement, and body hair glossiness of three animal groups were observed and recorded. The characteristic manifestations of the rat syndromes were also observed.
2. Evaluation of neurological deficits: A validated five-point scale was used to quantify the neurological deficit scores for all rats at 24 h postoperatively^23^. Specifically, a rat with no neurological deficit symptoms received a score of 0, a rat failing to completely stretch the left fore paw received a score of 1, a rat circling to the left received a score of 2, a rat falling to the left or rolling on the ground received a score of 3, and a rat showing no spontaneous activity with consciousness disorder received a score of 4.
3. Tail blood flow perfusion detection: The PeriCam PSI system (Perimed, Sweden) was used to detect blood perfusion in the rats’ tail tips. We focused the cursor on the 1 cm point at the tip of the rat tail, and then observed and recorded the tail blood perfusion. The laser blood perfusion speckle image was generated, and then the average blood perfusion of the tail tip of each group of rats was analyzed using PIMSoft software along with the PeriCam PSI system.
4. Detection of Whole Blood Viscosity (WBV), Plasma Viscosity (PV), and platelet Aggregation Rate (AR): Blood samples were extracted from the abdominal aorta of rats. The blood was then collected into one heparin anticoagulant tube and one sodium citrate anticoagulant tube. Subsequently, WBV, PV, and platelet AR were detected in rats using a fully automatic hemorheological analyzer (Beijing Succeeder Technology Inc., China) and a PL-12 platelet function analyzer (SINNOWA Medical Science and Technology Co., Ltd., China).
5. Measurement of the cerebral infarction area: We performed 2,3,5-Triphenyltetrazolium Chloride (TTC, Sigma, USA) staining to visualize the ischemic infarction area. All rat brains were sliced into 2-mm-thick coronal sections before incubating each slice in a 0.1% TTC solution at 37 °C for 30 min. The slices were then fixed in 4% paraformaldehyde. The infarction area was quantified using Image J software.
6. Hematoxylin–eosin staining (HE): Brain samples were swiftly extracted from all rats, followed by overnight fixation in 4% paraformaldehyde. Subsequently, the brains were dehydrated using graded alcohol and encased in paraffin wax. The 5 μm thick-paraffin-embedded brain tissue sections were then processed with the HE kit to observe the neuronal pathological changes.
7. Enzyme-Linked Immunosorbent Assay (ELISA): Cortical tissue samples were extracted from the rats’ ischemic hemisphere and the corresponding side in the NC group. The tissue samples were then incubated with an appropriate lysis buffer volume and mechanically processed using a cold grinder. The mixture was allowed to settle before obtaining the supernatant by centrifuging it at 3000 rpm for 10 min at 4 °C. The interleukin-6 (IL-6) expression level in the rat brain tissue was determined using an ELISA kit per the recommended protocol.

### Research on the biological basis of the disease-syndrome combination model through integrated transcriptomics and metabolomics analysis

Based on the established model, transcriptomic and metabolomic analyses were performed on the brain tissues of the three groups of rats to explore the biological mechanisms of the DS model.

#### RNA-seq-based transcriptomic study

##### RNA extraction, library construction and sequencing

Total RNA was extracted from the ischemic cortical tissue of rats using the Trizol Reagent (Invitrogen Life Technologies). Four samples were processed per group. A NanoDrop spectrophotometer (Thermo Scientific) was used to assess the concentration, quality, and integrity of the extracted RNAs. Three micrograms of RNA were used as input material for RNA sample preparations.

The RNA library was completed by Shanghai Personal Biotechnology Co. Ltd. A total RNA of ≥ 1 μg was selected, and cDNA was synthesized using the NEBNext Ultra II RNA Library Prep Kit (Illumina). The AMPure XP beads were used to screen cDNA fragments of around 400–500 bp, perform PCR amplification, and purify the PCR product, resulting in a library. Sequencing was performed using the NovaSeq 6000 platform (Illumina) after completing the library quality inspection.

##### Differential gene expression analysis

The image file was obtained after sequencing the sample on the machine, and the sequencing platform generated the original FASTQ data (Raw data). Quality checks were performed on raw data using FastQC v0.11.8. Reads that met the quality control (QC) standards for the rat reference genome were mapped using the HISAT2 aligner v2.0.5. The read count of the original expression level of each gene was obtained using HTSeq. Fragments Per Kilobase of transcript per Million fragments mapped (FPKM) were used to normalize expression levels to ensure comparability of gene expression levels between different genes and samples. Transcriptomic analysis was performed through Principal Components Analysis (PCA). Differential Gene (DG) expression analysis was performed using the DESeq2 package, with *P* < 0.05 and |log2FoldChange| > 1 as the screening conditions. Gene ontology (GO) and Kyoto Encyclopedia of Genes and Genome (KEGG) analyses of DGs were performed using the Database for Annotation Visualization and Integrated Discovery (DAVID).

#### Untargeted metabolomic study

##### Sample pretreatment

Sample preparation and liquid chromatography—tandem mass spectrometry (LC–MS/MS) detection were completed by Shanghai Personal Biotechnology Co. Ltd. Eight ischemic cortical tissue samples per group were thawed gradually at 4 °C. Subsequently, 1 mL of precooling methyl alcohol/acetonitrile/water (2:2:1, v/v) was added, and the mixture was sufficiently vortexed. After 30 min of low-temperature ultrasonic breakdown, the samples were centrifuged at 14,000×*g* for 20 min at 4 °C to precipitate the protein. The supernatants were collected, vacuum-dried, and kept at − 80 °C, awaiting further experiments. The material was then resolved in 100 μL acetonitrile/water (1:1, v/v), sufficiently vortexed, and centrifuged at 14,000 rpm, for 15 min at 4 °C. Following that, the supernatants were subjected to LC–MS/MS analysis.

##### LC–MS/MS analysis

Chromatographic separation was performed using an ACQUITY UPLC BEH C18 column (100 mm × 2.1 mm, 1.7 μm, Waters, USA) with a column temperature of 40 °C and a flow rate of 0.3 mL/min. The mobile phase A consisted of water with 0.1% formic acid, while mobile phase B was acetonitrile. The metabolites were eluted using the following gradient: 0–0.5 min, 5%B; 0.5–1.0 min, 5%B; 1.0–9.0 min, 5–100%B; 9.0–12.0 min, 100%B; 12.0–15.0 min, 5%B. The sample injection volume for each sample was 5 μL. Throughout the analysis, samples were kept in an autosampler at 4 °C. To avoid any impact from instrument signal fluctuations, samples were analyzed in random order. Quality control (QC) samples were inserted after each group of samples in the sample queue to monitor and assess system stability and the reliability of experimental data.

The MS conditions were as follows: Ion source: electrospray ionization (ESI); Samples were detected in both ESI positive and negative modes. Mass spectrum parameters: Ion source gas1 (Gas1): 60; Ion source gas2 (Gas2): 60; Curtain gas: 30; Source temperature: 320 °C; Spray Voltage (V): 3500 (positive ion), − 3500 (negative ion). In MS only acquisition, the instrument was set to acquire over the m/z range 60–1000 Da, product ion scan m/z range 25–1000 Da, MS scan accumulation time 0.20 s/spectra, product ion scan accumulation time 0.05 s/spectra. MS/MS is acquired using information dependent acquisition (IDA) with high sensitivity mode selected. The collision energy (CE) was fixed at 35 eV with ± 15 eV. Declustering potential (DP) was set as ± 60 V. IDA was set as follows: Exclude isotopes within 4 Da; Candidate ions to monitor per cycle: 6.

##### Data preprocessing and statistical analysis

The acquired LC–MS/MS raw data were preprocessed by Compound Discoverer 3.0 (Thermo Fisher Scientific) software, including peak extraction, peak alignment, peak correction, and normalization. A three-dimensional data matrix composed of sample names, peak information (including retention time and molecular weight), and peak areas was output. The structural identification of metabolites was conducted by using accurate mass matching (< 25 ppm) and MS/MS spectral matching, and searching through the self-built database in the laboratory, as well as other online databases such as Bio cyc, HMDB, Metlin, HFMDB, and Lipidmaps.

In the extracted ion features, only the variables having more than 50% of the nonzero measurement values in at least one group were kept. SIMCA-P 14.1 (Umetrics, Umea, Sweden) was used for Orthogonal partial least-squares-discriminant analysis (OPLS-DA). The differential metabolites (DMs) between groups were screened based on a threshold of variable importance on the projection (VIP) values obtained from the OPLS-DA model, where metabolites with VIP > 1.0 and *P* < 0.05 were considered DMs.

#### Gene–metabolite network construction

The DGs and DMs were entered into the “Network Analysis” module of the MetaboAnalyst platform to explore the transcriptome–metabolome biological connections. The Cytoscape software was utilized to visualize the “gene–metabolite” network.

### Statistical methods

Data management and statistical analyses were performed using GraphPad Prism software (San Diego, CA). The results are presented as Mean (M) ± Standard Deviation (SD). The independent sample *t*-test or one-way ANOVA was utilized for data analysis. Results with *P* < 0.05 were considered statistically significant, with *P* < 0.01 showing a highly significant difference.

### Ethical statement

The study was approved by Experimental Ethics Committee at Xiyuan hospital, China Academy of Chinese Medical Sciences (No. 2022XLC045-2), all methods were carried out in accordance with relevant guidelines and regulations. This study was carried out in compliance with the ARRIVE guidelines.

## Results

### General information and characteristic manifestations of the syndrome

Zero, one, and one deaths were reported in the control, disease, and DS groups, respectively. The NC group animals had a normal diet, free movement, good mental state, and slightly rough hair before sampling. Rats in the disease and DS groups exhibited a significant decrease in activity, reduced food intake, loose and matte hair, decreased body mass, and decreased energy levels. In TCM theory, it is believed that blood stasis and toxin often damage body functions, leading to symptoms such as mental depression and fatigue. The DS group rats showed more significant mental distress, preferring to curl up in a corner with their hair in a ‘burst’ state, and were also less resistant when touched.

**Figure 2:**
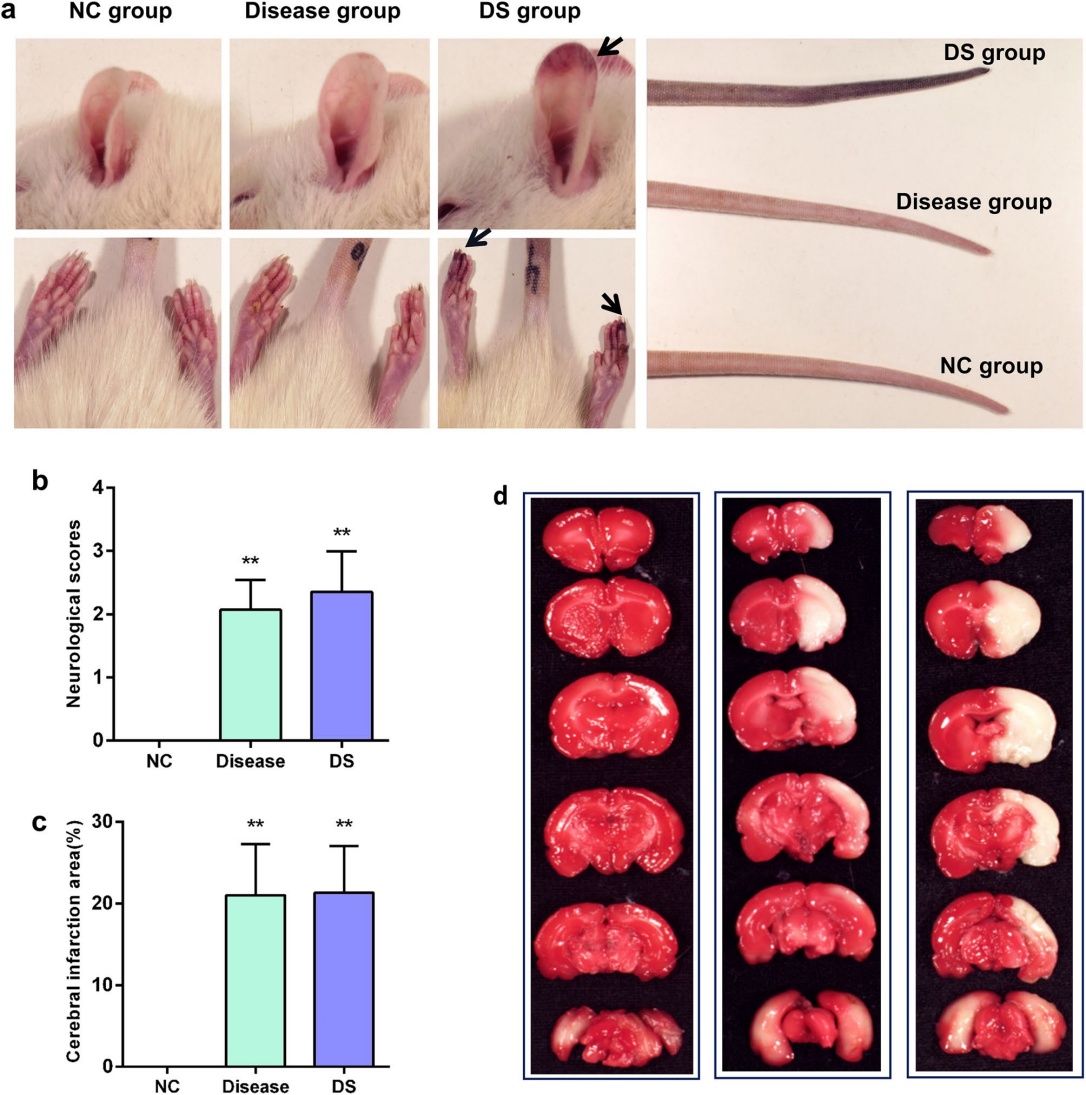
(**a**) Syndrome characteristics of rats in each group after modeling. (**b**) Neurological score was measured 24 h postoperatively. **p < 0.01 against the NC group. (**c**) Measurement of the cerebral infarction area (n = 6/group). **p < 0.01 against the NC group. (**d**) Cerebral infarction area was assessed through TTC staining 24 h post-surgery.

Furthermore, after modelling, the DS group animals showed swollen and black purple claw nails, as well as noticeable purple and dark auricular veins and a “black tail” state at the tail. The other rat groups showed no significant changes in characterization (Fig. 2a). According to the TCM basic theory, the accumulation of blood stasis and toxin in the body can cause poor blood circulation, or even damage the blood vessels, leading to ecchymosis on the surface of the body, local tissue swelling or necrosis. Therefore, based on these manifestations, the DS group exhibited more obvious syndrome characteristics.

### Comparison of neurological deficits and the cerebral infarction areas

We evaluated the degree of neurological deficits in each group of rats 24 h post-surgery. The neurological function scores of the disease model and the DS model groups were significantly higher than those of the NC group (*P* < 0.01) (Fig. 2b). On the other hand, the cerebral infarction area was assessed using TTC staining at 24 h postoperatively. The disease model and DS groups had a significantly greater infarction size than the NC group (*P* < 0.01) (Fig. 2c,d). Furthermore, there was no significant difference between the disease and DS groups (Supplementary Information 1).

### Comparison of tail blood flow perfusion

**Figure 3:**
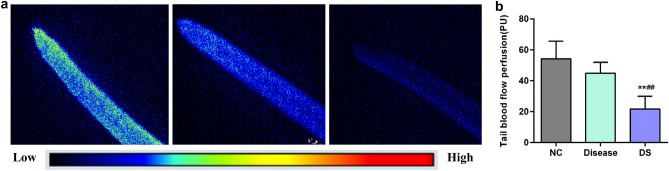
(**a**) Representative images of tail blood flow perfusion in each rat group. (**b**) Comparison of tail blood flow perfusion in each rat group (n = 7). **p < 0.01 against the NC group. ^##^*p* < 0.01 against the disease group.

The blood perfusion at the tail end of rats usually refers to the blood flow in a specific area of the tail. A state of blood stasis may affect the blood perfusion at the tail of rats, causing it to decrease or be blocked. The number of warm tone pixels was positively correlated with the richness of blood flow per unit area in laser speckle imaging. The tail-end blood flow perfusion of the NC group was abundant compared to that of the DS group, which was significantly lower (*P* < 0.01). On the other hand, although the tail-end blood flow perfusion in the disease group exhibited a decreasing trend compared to the control group, there was no statistical significance (Fig. 3a,b).

### Comparison of WBV, PV, and platelet AR

Abnormal changes in blood rheology such as fluidity and viscosity are important pathological mechanisms that progress from blood stasis to the coexistence of blood stasis and toxin. Blood rheology reflects the flow and viscosity of blood, serving as a crucial indicator of the body's blood stasis condition^24^. Compared to the NC group, the WBV at the median shear rate was significantly higher in the disease and DS groups (*P* < 0.05, *P* < 0.01). Notably, the DS group had a significantly higher WBV at the low/median/high shear rate (*P* < 0.05, *P* < 0.01). Consistent with the WBV results, the PV was markedly higher in the disease and DS groups than the NC group (*P* < 0.01). Furthermore, the DS group had a significantly higher PV than the disease group (*P* < 0.01). Additionally, the maximum and average platelet ARs were significantly higher in the disease and DS model groups than the control group (*P* < 0.01). However, there were no significant statistical differences between the disease and DS groups in platelet ARs. These findings show that the DS model rats exhibited more severe blood stasis state. These results are summarized in Tables 1 and 2.

**Table 1:** Comparison of WBV and PV in each rat group (M ± SD, n = 10).

Group	WBV (mPa's)			PV (mPa's)
	Low shear rate	Median shear rate	High shear rate	
NC group	9.60 ± 1.24	4.61 ± 0.47	4.08 ± 0.42	1.18 ± 0.09
Disease group	11.39 ± 2.25	5.34 ± 0.63*	4.35 ± 0.59	1.28 ± 0.14**
DS group	11.64 ± 1.66*	5.52 ± 0.50**	4.75 ± 0.43*	1.40 ± 0.07**^##^

*p < 0.05, **p < 0.01 vs. the NC group. ^##^*p* < 0.01 vs. the disease group.

**Table 2:** Comparison of platelet AR in each rat group (M ± SD, n = 10).

Group	Platelet AR(%)	
	Maximum aggregation rate	Average aggregation rate
NC group	36.85 ± 13.26	29.98 ± 10.38
Disease group	64.70 ± 11.02**	51.03 ± 12.64**
DS group	70.16 ± 7.91**	57.93 ± 11.43**

**p < 0.01 vs. the NC group.

### Comparison of IL-6 expression levels in brain tissue

**Figure 4:**
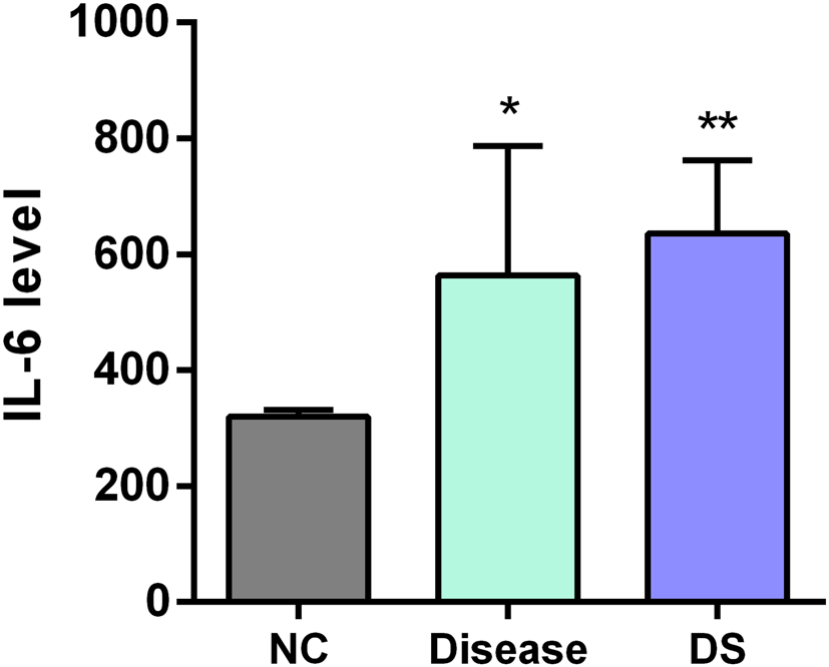
ELISA was used to determine IL-6 expression in ischemic cortical tissue. *p < 0.05, **p < 0.01 vs*.* the NC group.

Whether in the pathogenesis of cerebral infarction or in the formation process of BST syndrome, there will be an increase in inflammatory cytokines. The expression level of IL-6 was significantly higher in the brain tissue of rats in the disease and DS groups compared with the levels in the control group rats (*P* < 0.05, *P* < 0.01). There was no significant differences between the disease and DS groups (Fig. 4).

### Comparison of HE staining examination

**Figure 5:**
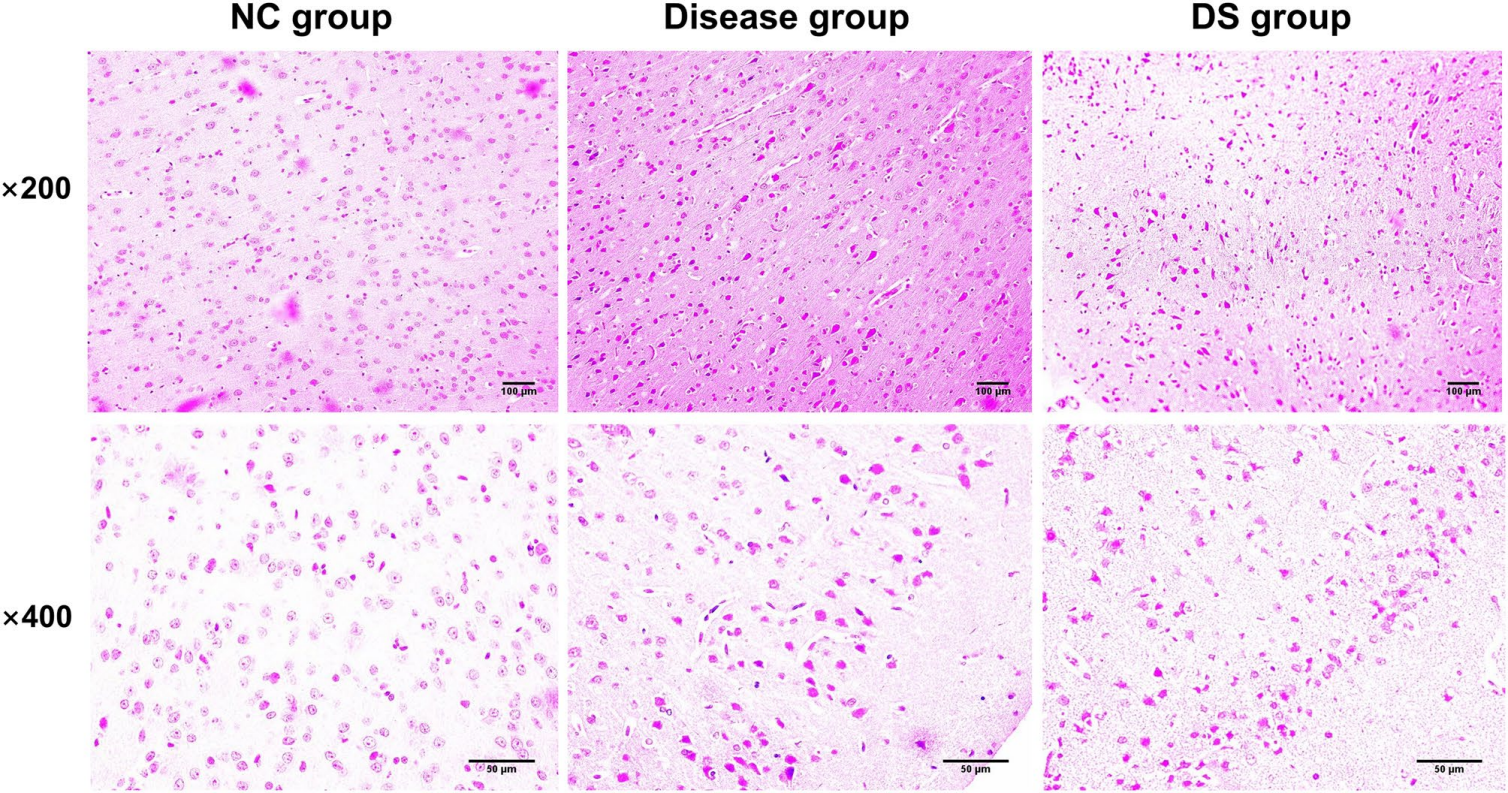
Representative images of histopathological changes in the brain tissues in three rat groups captured under a 200× and 400× light microscope.

Pathological and morphological changes in brain tissue were observed through HE staining. Neuronal cells in the cortex of the NC group rats were arranged neatly, with normal neuronal morphology (Fig. 5). There were no pathological changes such as degeneration or necrosis in the NC group. On the other hand, the disease and DS groups showed noticeable pathological alterations, including disordered cell arrangement, loose structure, common neuronal degeneration and necrosis, nuclear pyknosis, and glial cell proliferation.

### Transcriptomic characteristics of the IS-BST syndrome

**Figure 6:**
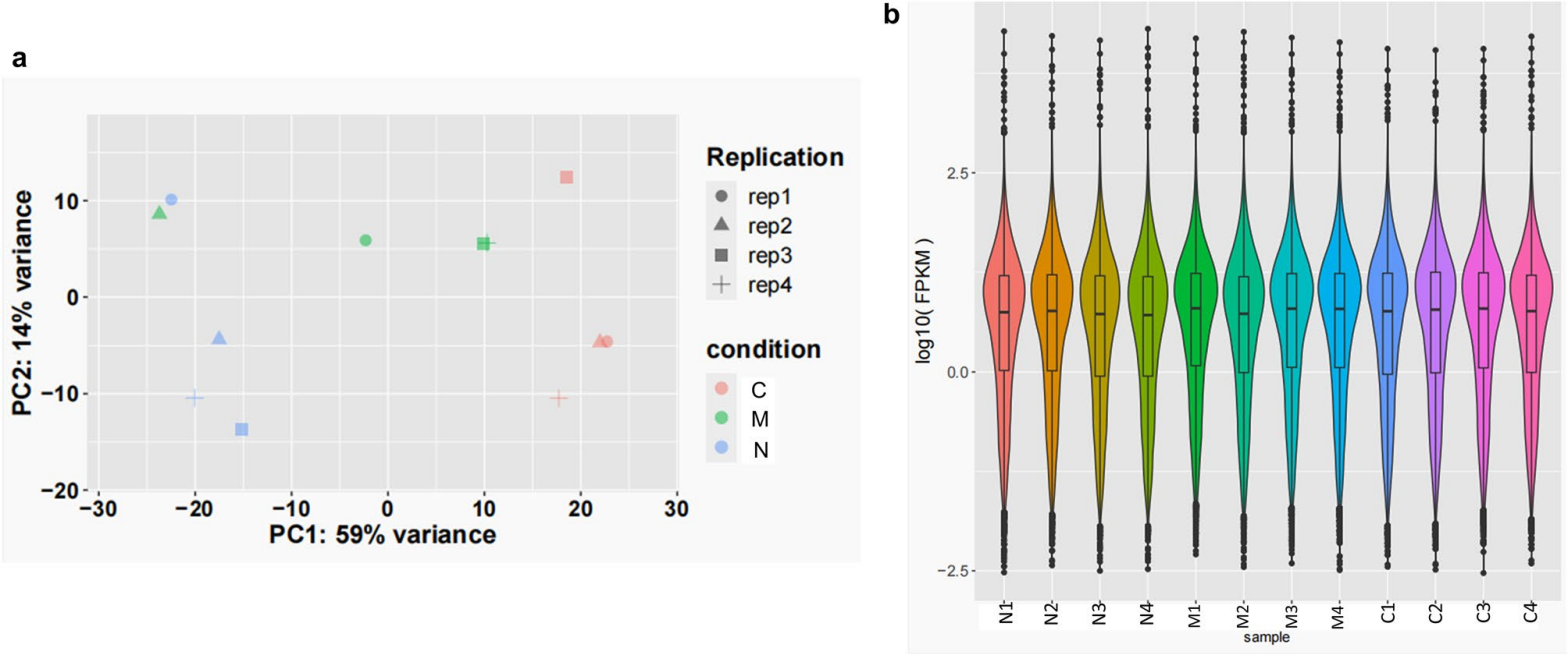
(**a**) Principal component analysis: The horizontal axis represents the first principal component, and the vertical axis represents the second principal component; Different colors represent different groups, and different shapes represent different samples. (**b**) FPKM density distribution. *N* the NC group, *M* the disease group, *C* the DS group.

PCA analysis revealed a clear distinction between the three groups along the first principal component with a 59% explained variance (Fig. 6a). The FPKM density distribution can intuitively reflect the general patterns and characteristics of RNA seq data at the quantitative level. As shown in Fig. 6b, the FPKM homogeneity was good across individual samples, suggesting that the quality of each sample was good and reliable.

**Figure 7:**
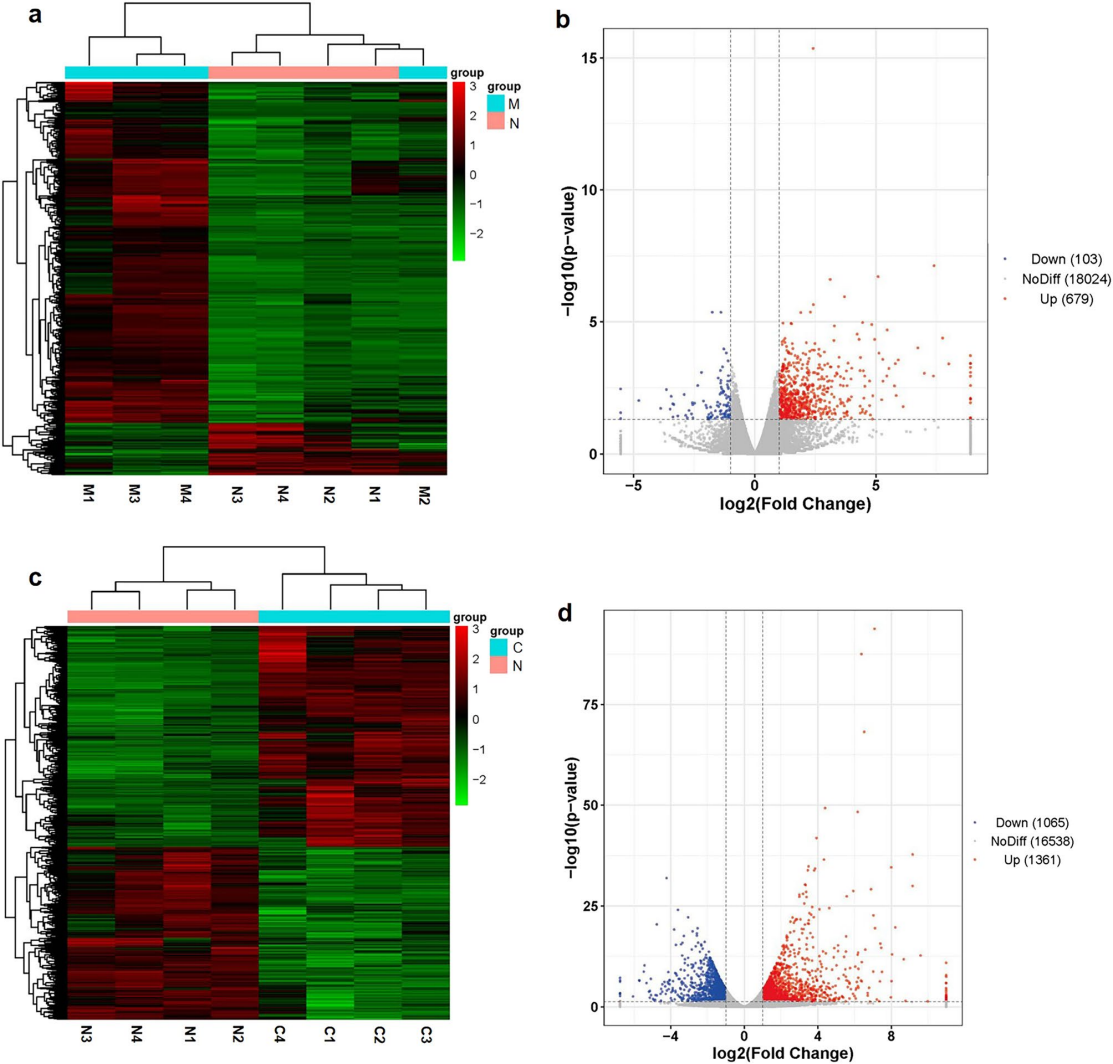
(**a**) Heat map of DGs between the NC and disease groups. (**b**) Volcano plot of DGs between the NC and disease groups. (**c**) Heat map of DGs between the NC and DS groups. (**d**) Volcano plot of DGs between the NC and DS groups. *N* the NC group, *M* the disease group, *C* the DS group.

In total, 782 DGs were identified between the disease group and NC groups, with 679 DGs upregulated and 103 DGs downregulated. The heat and volcano maps showed the expression of DGs between Disease group and NC group (Fig. 7a,b). In addition, 2426 DGs were screened between the DS and NC groups, of which 1361 and 1065 DGs were upregulated and downregulated, respectively. Figure 7c,d show the heatmap and volcano plot for all DGs, respectively (Supplementary Information 2, 3, 5, 6).

**Figure 8:**
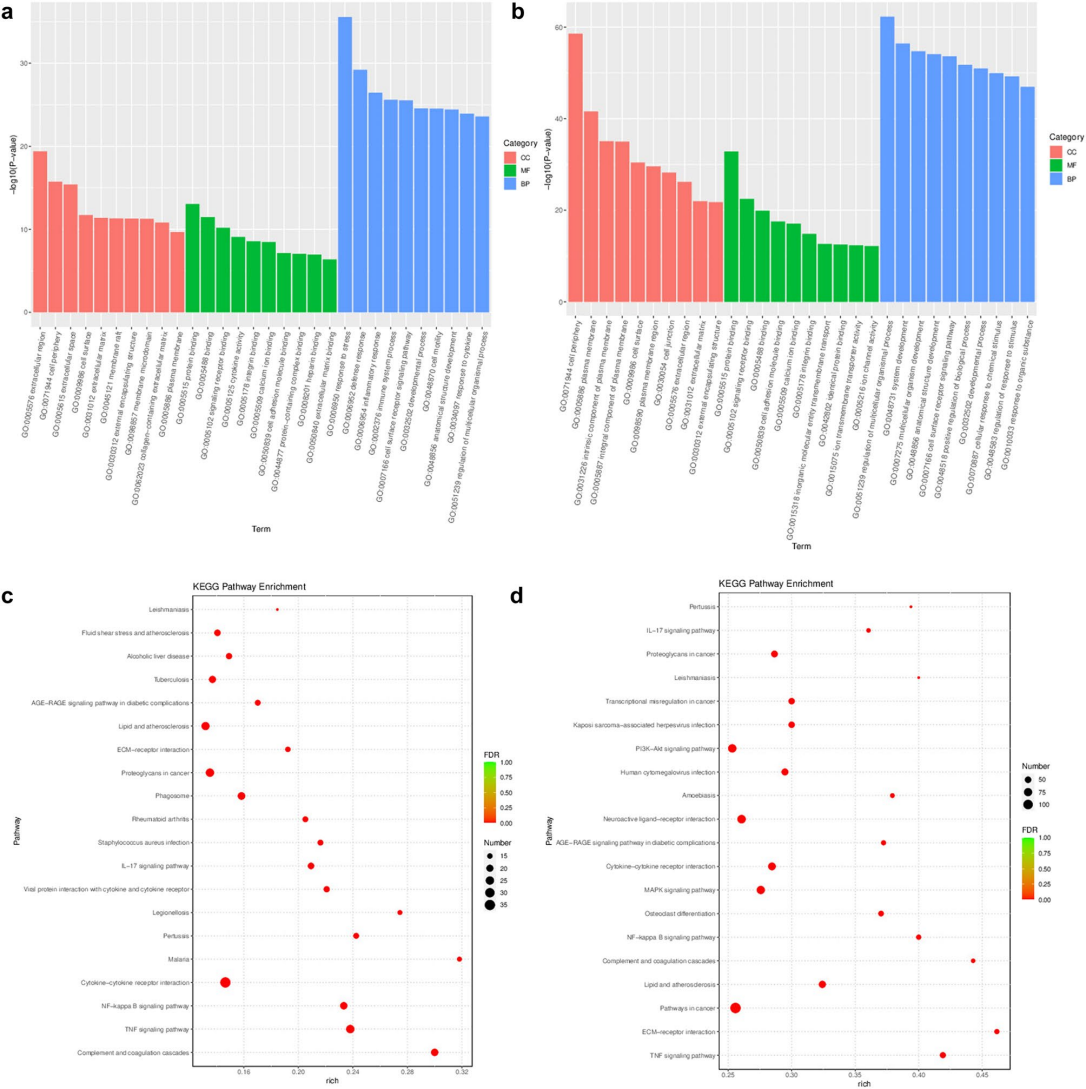
(**a**) GO enrichment analysis of the DGs in disease group (red, green, and blue represent the CC, MF, and BP terms, respectively). (**b**) GO enrichment analysis of the DGs in DS group. (**c**) Bubble chart showing the top 20 pathways of DGs between the NC and disease groups. (**d**) Bubble chart showing the top 20 pathways of DGs between the NC and DS groups.

Subsequently, the Gene Ontology (GO) function and Kyoto Encyclopedia of Genes and Genomes (KEGG) pathway enrichment analyses were performed on the DGs. The GO analysis comprises Biological Processes (BP), Cellular Components (CC), and Molecular Functions (MF). The top ten significant enrichment terms of BP, CC, and MF with the highest gene counts were visualized in a bar chart. Most of the enriched BP terms of the DGs in the disease model were mainly associated with response to stress, defense response, and inflammatory response. In the CC domain, the DGs of the disease model were mainly involved in the extracellular region, cell periphery, and extracellular space; further, a strong increase in the genes was mainly involved in protein binding, binding, and receptor binding (Fig. 8a). The DGs of DS model were primarily enriched in regulation of multicellular organismal process, system development, multicellular organism development, and other biological processes; cell periphery, plasma membrane, intrinsic component of plasma membrane, and other cellular components; protein binding, receptor binding, binding, and other molecular functions (Fig. 8b).

The bubble diagram showed the top 20 significant enrichment potential pathways with the highest gene counts. The results revealed that the DGs of disease group were mainly enriched in complement and coagulation cascades, TNF signaling pathway, NF-kappa B signaling pathway, cytokine-cytokine receptor interaction, etc. (Fig. 8c). The DGs of DS group were mainly enriched in the TNF signaling pathway, the ECM-receptor interaction pathway, cancer pathways, the lipid and atherosclerosis pathway, and complement and coagulation cascades, among other pathways (Fig. 8d).

### Metabolomic characteristics of the IS-BST syndrome

**Figure 9:**
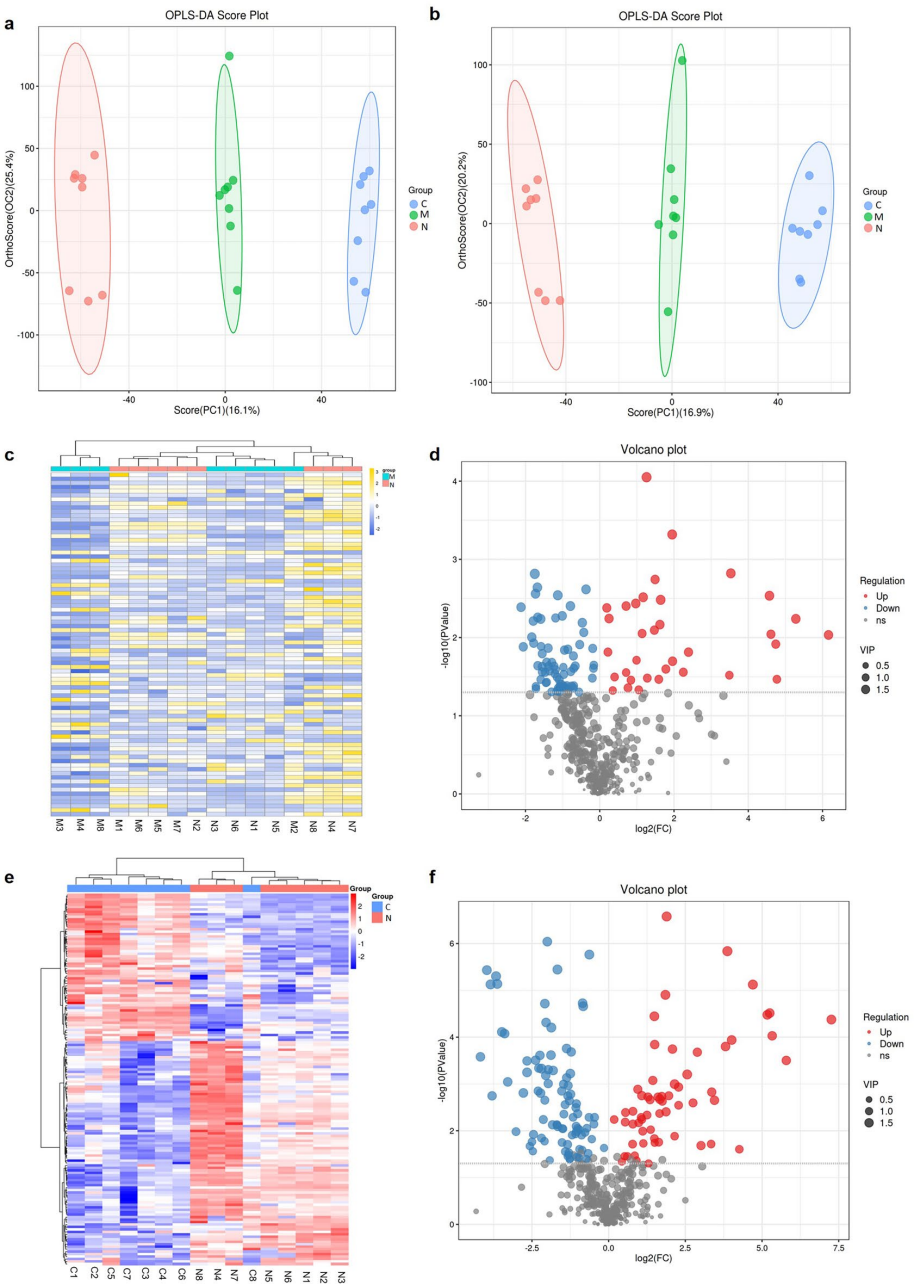
(**a**) OPLS-DA analysis of three groups: Positive ion mode. (**b**) OPLS-DA analysis of three groups: Negative ion mode. (**c**) Heat map of DGs between the NC and disease groups. (**d**) Volcano plot of DGs between the NC and disease groups. (**e**) Heat map of DGs between the NC and DS groups. (**f**) Volcano plot of DGs between the NC and DS groups. *N* the NC group, *M* the disease group, *C* the DS group.

Herein, 14,623 metabolites were discovered, of which 473 were annotated in the online databases and self-built database in the laboratory. The DMs were generated through OPLS-DA analysis using the SIMCA software, with VIP > 1 and *P* < 0.05 as the screening conditions. The OPLS-DA score revealed that the NC, disease, and DS groups exhibited a clear trend of separation (Fig. 9a,b). A total of 102 metabolites were identified between the disease group and NC groups, with 34 DMs upregulated and 68 DMs downregulated. These 102 metabolites are visualized by heat maps and volcano maps (Fig. 9c,d). Compared with the NC group, 151 metabolites were altered in the DS group, of which 60 and 91 DMs were screened between the two groups and established to be upregulated and downregulated, respectively. Figure 9e,f show the heatmap and volcano plot for all DGs, respectively (Supplementary Information 4 & 7).

**Figure 10:**
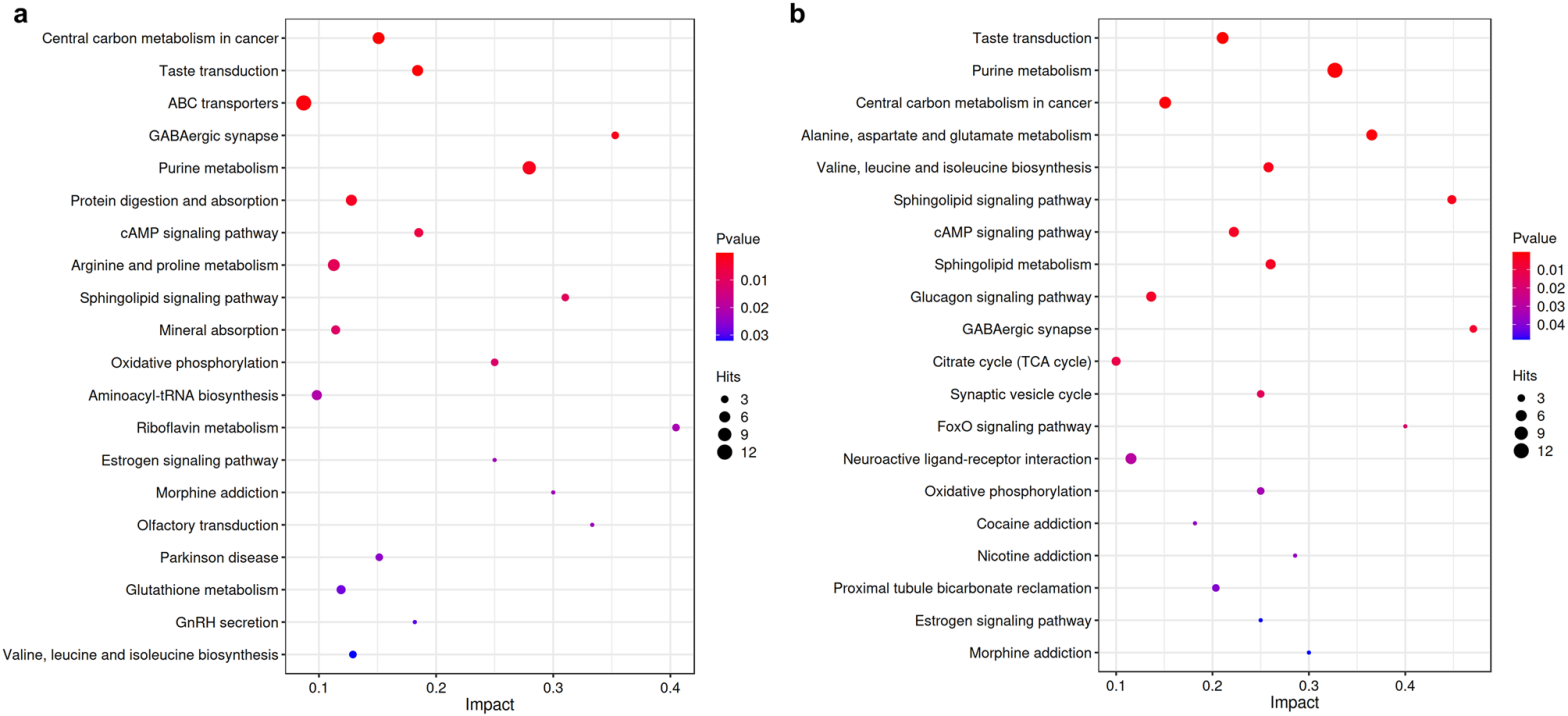
(**a**) Bubble chart showing the top 20 pathways of DMs between the NC and disease groups. (**b**) Bubble chart showing the top 20 pathways of DMs between the NC and DS groups.

Key pathways implicated in the disease model and the DS model were identified by metabolic pathway analysis. Several metabolic pathways related to disease group were identified, including central carbon metabolism in cancer, taste transduction, ABC transporters, GABA ergic synapse, and purine metabolism (Fig. 10a). In addition, several metabolic pathways such as the taste transduction, purine metabolism pathway, central carbon metabolism in cancer, alanine, aspartate, and glutamate matabolism, valine, leucine and isoleucine biosynthesis, and so on were significantly associated with the DS group (Fig. 10b).

### DGs–DMs interaction analysis

**Figure 11:**
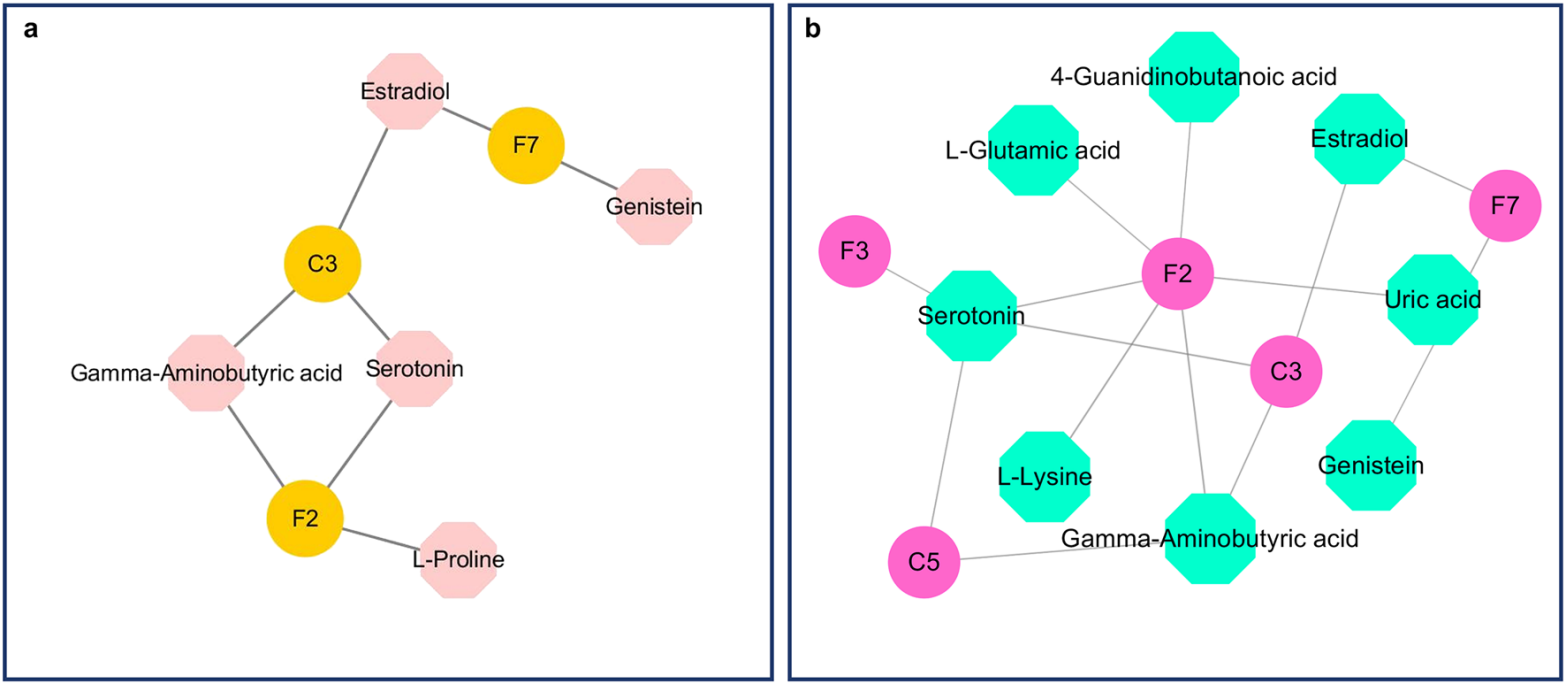
(**a**) The “gene–metabolite” network of the disease model. (**b**) The “gene–metabolite” network of the DS model.

By integrating transcriptomics and metabolomic data, we established a “gene–metabolite” network for the disease model and DS model. As shown in Fig. 11a and Table 3, the “gene–metabolite” network of the disease model comprised three mRNAs (C3, F2, and F7) and five metabolites (serotonin, gamma-aminobutyric acid, genistein, estradiol and l-proline). C3 was the most relevant gene with a degree and betweenness of 3 and 12.5, respectively. Estradiol was the metabolite most related to genes, with a degree and betweenness of 2 and 10, respectively.

**Table 3:** The “gene–metabolite” network of the disease model.

Gene/metabolite	Degree	Betweenness	Up/down
C3	3	12.5	Up
F2	3	6.5	Up
Estradiol	2	10	Down
F7	2	6	Up
Serotonin	2	4	Up
Gamma-aminobutyric acid	2	4	Down
Genistein	1	0	Up
l-Proline	1	0	Down

Up: Compared with the NC group, the upregulated genes or metabolites in the disease group. Down: Compared with the NC group, the upregulated genes or metabolites in the disease group.

Five mRNAs (F2, C3, F7, C5, and F3) and eight metabolites (serotonin, gamma-aminobutyric acid (GABA), estradiol, l-glutamic acid, l-lysine, genistein, uric acid (UA), and 4-guanidinobutanoic acid) made up the “gene–metabolite” network of the DS model. The most significant metabolite was serotonin, which had degrees and betweennesses of 4 and 25.5, respectively. F2 was the gene most closely associated with metabolites, with betweenness and degree of 6 and 38.67, respectively. Furthermore, F2 was linked to serotonin, l-glutamic acid, 4-guanidinobutanoic acid, l-lysine, GABA, and UA (Fig. 11b and Table 4).

**Table 4:** The “gene–metabolite” network of the DS model.

Gene/metabolite	Degree	Betweenness	Up/down
F2	6	38.67	Up
Serotonin	4	25.5	Up
C3	3	27.67	Up
Gamma-aminobutyric acid	3	14.5	Down
Estradiol	2	20	Down
F7	2	11	Up
C5	2	0.67	Up
F3	1	0	Up
l-Glutamic acid	1	0	Up
l-Lysine	1	0	Down
Genistein	1	0	Up
Uric acid	1	0	Up
4-Guanidinobutanoic acid	1	0	Up

Up: Compared with the NC group, the upregulated genes or metabolites in the DS group. Down: Compared with the NC group, the upregulated genes or metabolites in the DS group.

## Discussion

Ischemic Stroke (IS) is a common illness with profound health implications. In recent years, TCM, which has special benefits regarding IS treatment, has received substantial attention. Syndrome differentiation-based treatment is the fundamental principle of TCM in understanding and treating diseases^25^. Currently, the ‘disease-syndrome combination’ is not only a clinical stroke diagnosis and treatment approach, but also a highly consensus research model. The “disease-syndrome combination” animal model is a disease model-based animal model with good reliability and stability. At the same time, the introduction of the ‘syndrome’ concept in TCM has proven to be valuable in reflecting the phased and dynamic changes in disease characterizations in TCM. Specifically, it serves as a platform and conduit for researching clinical diseases in the field of TCM.

Herein, on the basis of our preliminary work, we used a multi-factor combination to construct an animal IS model with blood stasis and toxin syndrome, and explored the biological mechanisms underlying the model through transcriptomic and metabolomic analyses. Our findings hold academic significance as they contribute to the exploration of therapeutic principles underlying TCM formulae and the development of precision medicine for IS treatment.

### Multiple evaluation indicators show that the combination of carrageenan and active dry yeast along with MCAO, can be used to successfully establish an IS-BST animal model

Motor dysfunction is one of the main IS manifestations. Herein, rats in both the disease and DS groups showed left limb hemiplegia, with decreased neurological function scores. The cerebral ischemic injury in rats was further confirmed by TTC staining. Compared to the disease model, the DS model included an additional TCM-BST syndrome based on motor dysfunction. The TCM basic theory posits that blood stasis and toxin damage the veins and collaterals, which in turn causes blood to overflow outside the veins and accumulate under the skin, resulting in bruises and ecchymosis on the skin. Therefore, apart from symptoms such as mental distress, decreased activity, reduced food intake, and rough hair, the characteristics of the DS group also included ecchymosis on rats’ ears and claws and thrombi in their tails. Additionally, rats in the DS group also showed a significant decrease in tail blood flow perfusion.

The BST syndrome consists of two syndrome elements: blood stasis and toxin. Pertinent modern studies often interpret blood stasis as abnormal hemorheology, aberrant platelet aggregation function, microcirculation disorders, and so on^26^. Inflammatory responses are often used as a common indicator for evaluating toxins and pathogens^11^. At the same time, thrombosis and inflammatory responses are highly connected mechanisms that promote neuronal damage after ischemia in the complex IS pathological process^27^. Our findings revealed that WBV (low, medium, and high shear), PV, and platelet AR were significantly higher in the DS group than the NC group. It is well-documented that IL-6 is an important inflammatory response marker post-IS^28^. In this study, the IL-6 expression levels in brain tissue were significantly increased in both the disease and DS model groups.

Histopathology can objectively reflect model establishment success and disease severity. Herein, the disease and DS groups showed severe pathological damage, but with no histopathological differences.

**Figure 12:**
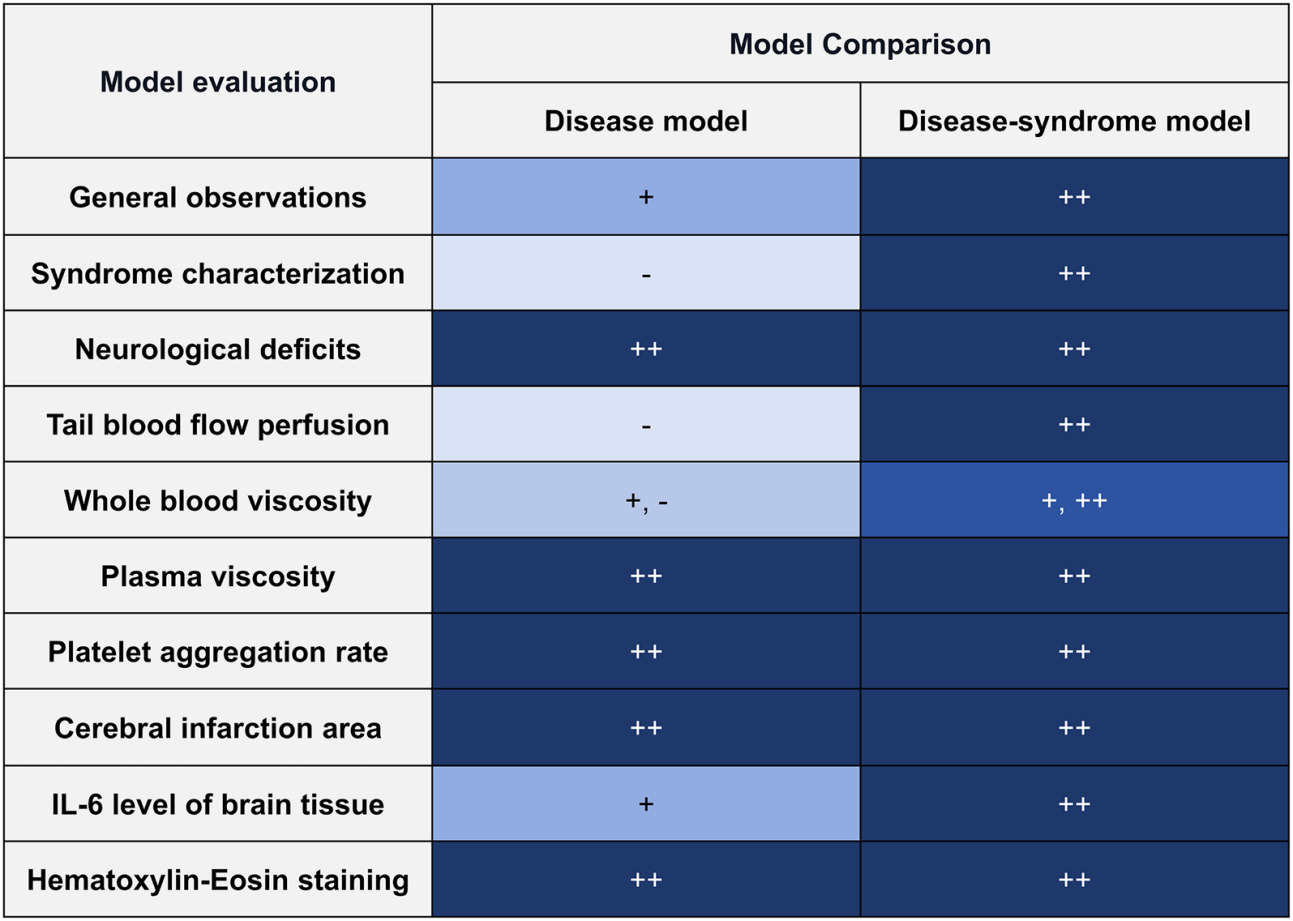
+ denotes *P* < 0.05 vs. NC group, ++ denotes *P* < 0.01 vs. NC group. The DS group presents more severe disease and syndrome characteristics, making it the preferred standard for constructing the IS-BST syndrome model.

Based on the above-mentioned findings, we inferred that the DS group had more stable IS characteristics and the blood stasis and toxin syndrome, implying that it is a preferable standard for constructing an IS-related blood stasis and toxin accumulation animal model (Fig. 12).

### Transcriptomic and metabolomic strategies could reveal the biological basis of the IS-BST syndrome

#### Transcriptomics analysis and metabolomics analysis

In the transcriptomics study, 782 mRNAs were identified as DGs for the disease. They were mainly enriched in complement and coagulation cascades, TNF signaling pathway, NF-kappa B signaling pathway, cytokine–cytokine receptor interaction, etc. A total of 2426 mRNAs were screened as DGs for the DS model. They were enriched in the TNF signaling pathway, the ECM-receptor interaction pathway, cancer pathways, the lipid and atherosclerosis pathway, and complement and coagulation cascades, etc.

In the enrichment analysis of differential genes between the disease and DS models, the top 20 enriched pathways indicate that atherosclerosis, thrombosis, and inflammatory response are the main relevant pathways. Lipid and atherosclerosis as well as fluid shear stress and atherosclerosis are two of the signal pathways linked to atherosclerosis. Thrombosis-related signaling mechanisms include coagulation cascades and complement. The TNF signaling pathway, NF-kappa B signaling pathway, MAPK signaling pathway, IL-17 signaling pathway, etc. are among the signal pathways linked to the inflammatory response.

In the metabonomics study, 102 metabolites were identified as DMs for the disease. They were enriched in the GABAergic synapse, purine metabolism, protein digestion and absorption, cAMP signaling pathway, etc. A total of 151 metabolites were identified as DMs for the DS model. They were enriched in the alanine, aspartate and glutamate metabolism, valine, leucine and isoleucine biosynthesis, sphingolipid signaling pathway, cAMP signaling pathway, etc. The DS model is established on the basis of the disease model, so there are also some common metabolic pathways between the DS model and the disease model, such as purine metabolism, sphingolipid metabolism, cAMP signaling pathway, and so on.

### Gene–metabolite network analysis

#### Coagulation and complement cascade reaction and the IS-BST syndrome

However, single omics studies are difficult to comprehensively and systematically decipher the regulatory mechanisms of complex pathological processes. Herein, we integrated and analyzed the transcriptomic and metabolomic research findings to construct a “gene–metabolite” network. There are common signatures in the “gene–metabolite” network of the disease and DS models.

Prothrombin (F2), tissue factor (F3), and coagulation factor VII (F7) are the key coagulation elements in the coagulation system. Prothrombin (F2) is a thrombin precursor that exists in an inactive form in the bloodstream. A series of enzyme cascade reactions are triggered when blood vessels are injured, leading to the conversion of F2 into active thrombin. Active thrombin, as a strong activator, then converts fibrinogen into fibrin, promoting blood clot formation^29^. On the other hand, F3 mainly exists on the damaged vascular intima and tissue cells. It binds to F7 in the plasma when tissue damage occurs, triggering a coagulation cascade reaction^30^. Under the synergistic effect of F2, F3, and F7, blood stasis can cause endothelial damage and promote intravascular thrombosis. In this study, the levels of F2 and F7 were significantly elevated in the brain tissues of the disease model and DS model rats, and the levels of F3 were also significantly increased in the brain tissues of the DS model rats. This indicates that the IS-BST rats exhibit stronger coagulation features.

Various components of the complement system and coagulation system interact, activate, and regulate each to synergistically respond to host defense and damage repair. Complement component C3 (C3) is one of the most abundant components in the complement system, which can directly bind to platelets, fibrin, and molecules on the cell surface, thereby promote the coagulation process and thrombosis^31,32^. In addition, C3 is also a major participant in the initiation of inflammatory response in the central nervous system diseases^33,34^. Inhibiting C3 activity can alleviate the inflammatory response and decrease the volume of cerebral infarction in MCAO mice^35^. A significant increase in serum C3 levels in patients with ischemic stroke has been linked to poor clinical outcomes^36^. C5 is another key component of the complement system, which promotes the migration of neutrophils and monocytes to the injured site and enhances the release of inflammatory factors^37,38^. The expression of C5 was also upregulated in the brain after ischemic stroke, and the inhibition of C5 was found to significantly reduce infarct volumes and improve neurological scores^39^. In this study, C3 was significantly upregulated in the brain tissues of the disease model rats, while in the DS model, apart from upregulation of C3, C5 was also significantly upregulated, indicating a more pronounced inflammatory response in the IS-BST.

Although the complement and coagulation system are independent of each other, they closely function together, synergistically participating in key pathways such as thromboinflammatory response^40,41^. The “gene–metabolite” regulatory network diagram presented in this study indicates high correlation of these genes, especially in the DS model, suggesting that the biological basis for the interaction between blood stasis and toxin involves the complement and coagulation cascade reactions.

#### AA metabolism and IS-BST syndrome

In the “gene–metabolite” networks of the two models, there are some common metabolites, including serotonin, estradiol, gamma-aminobutyric acid (GABA), and genistein. Serotonin, also known as 5-hydroxytryptamine, is an indolamine with vasoconstrictive and aggregating properties. Researchers have demonstrated that serotonin can promote the development of platelets and increase procoagulant activity^42^. Researches indicate that acute ischemic stroke patients taking selective serotonin reuptake inhibitors can improve clinical recovery, with mechanisms including stimulating neurogenesis, anti-inflammation, and improving cerebral blood flow^43,44^. Estrogen is a lipophilic steroid hormone that exerts its functions by binding to estrogen receptors (ER). Estrogen receptors are present in various tissues, including brain parenchyma^45^. Research indicates that estrogen, especially estradiol, can mitigate brain damage caused by ischemic stroke by regulating immune cell responses^46^. GABA is considered as an inhibitory transmitter that can inhibit neuronal excitation and reduce neuronal damage caused by excitatory glutamate following cerebral ischemia^47^. In this study, serotonin was upregulated in the brain tissues of the disease model rats, while the levels of estradiol and GABA were downregulated.

In this study, l-proline is an unique metabolite in the disease model. A study has shown that five metabolites, including proline, are common in both animal models of ischemic stroke and clinical patients^48^. There may be a certain link between l-proline and ischemic stroke, but the specific mechanism of action still needs further research and exploration.

Studies have indicated that impaired amino acid metabolism is associated with the development of ischemic stroke and BST syndrome^13,49^. In the “gene–metabolite” regulatory network of DS model, the differential metabolites are mainly related to amino acid-related metabolism. In addition to serotonin and GABA, l-glutamic acid (Glu), l-lysine, and 4-guanidinobutyric acid also participate in amino acid metabolic pathways. Glu is the most abundant free amino acid in the brain and the main excitatory neurotransmitter in the brain. In cerebral ischemia, glu-mediated excitatory toxicity is an important mechanism leading to the occurrence of neuronal death and brain injury^50^. Lysine is an essential alkaline amino acid that can pass through the blood–brain barrier and provide the necessary energy for the repair and normal functioning of physiological activities of nerve cells. Oral administration of lysine was found to reduce the area of cerebral infarction in rats and alleviate brain edema^51^. 4-Guanidinobutyric acid is a metabolite in the process of converting arginine to GABA, and its reduced content may lead to a decrease in GABA^52^.

In brain tissue samples from the BST group, serotonin and l-glutamic acid were increased, while GABA and l-lysine were decreased. Moreover, the content of 4-Guanidinobutyric acid was observed to increase significantly. Regarding the inconsistent expression trends between 4-Guanidinobutyric acid and GABA, we hypothesized that the conversion of arginine to GABA involves multiple enzymatic reactions and intermediate products, with 4-guanidinobutyric acid being just one of them. Although changes in the content of 4-guanidinobutyric acid may affect the levels of its subsequent metabolites, it is not the sole factor determining the concentration of GABA. Furthermore, more replicated experiments are needed to verify the expression level of 4-guanidinobutyric acid in the brain tissue of DS model rats.

#### F2 (thrombin)-glutamate and blood stasis—toxin

**Figure 13:**
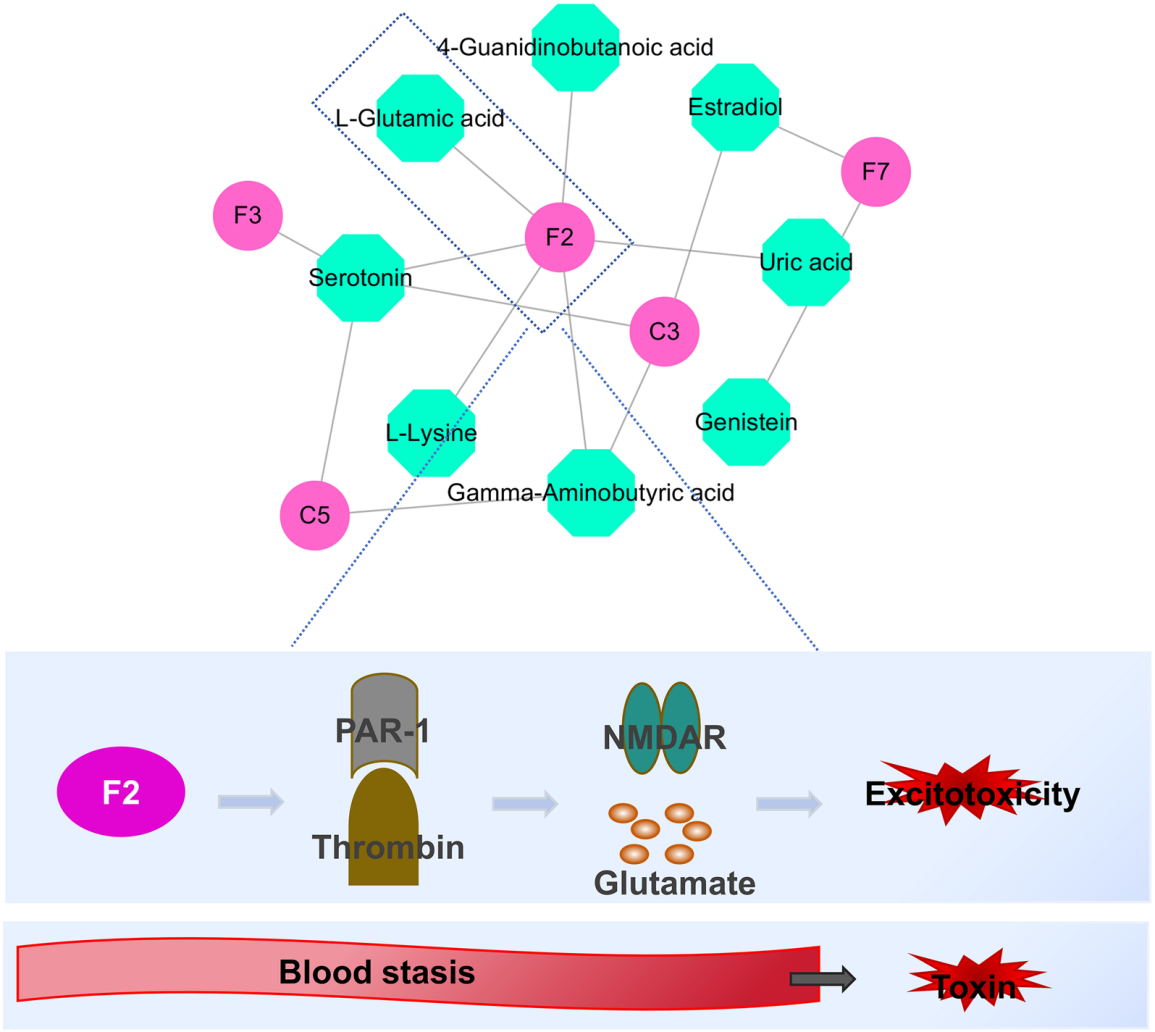
F2 (thrombin)-glutamate and blood stasis—toxin.

From the DS model “gene–metabolite” regulatory network, we found that F2 is the core gene with the highest degree of correlation. Thrombin, a serine protease, is encoded by the F2 gene. During cerebral ischemia, thrombin levels are elevated, which positively correlate with the infarct size^53,54^. High levels of thrombin has been linked to the occurrence of neurotoxicity^55^. Thrombin can cause blood–brain barrier disruption, increase endothelial permeability and damage to the brain tissue^56^. It has been demonstrated that thrombin stimulates NMDAR potentiation by activating its receptor PAR-1 (protease activator receptor-1), inducing a glutamate-mediated excitotoxicity^57,58^. As shown in the “gene–metabolite” network, l-glutamic acid is one of the downstream metabolites of F2. As we mentioned earlier, prolonged blood stasis leads to the production of toxins. To some extent, there is a strong similarity between “F2 (thrombin)–NMDAR/glutamate” pathway and the process of blood stasis brewing poison (Fig. 13). Therefore, we we have reasons to believe that the DS model “gene–metabolite” network can not only explain the pathogenesis of the disease, but also elucidate the biological significance of BST syndrome to a certain extent. However, our findings are based on animal research, and hence they may be somewhat different from actual clinical observations. Future research is necessary to validate these findings on IS-BST syndrome clinical patients.

## Conclusions

In this study, we constructed an animal model of IS-BST syndrome and established a model evaluation system that includes macroscopic characterization, microscopic indicators, and pathological morphology. It can be used to study conditions combining a disease and syndrome. By integrating transcriptomics and metabolomics research results, we found that IS-BST exhibits more prominent characteristics of coagulation and complement cascade reactions, as well as amino acid metabolism disorders. The “F2 (thrombin)-NMDAR/glutamate” pathway we inferred from the “gene–metabolite” regulatory network provides a clear direction for our subsequent pharmacological research. In conclusion, the IS-BST model aligns with TCM theories in understanding diseases and syndromes. It will help promote innovative research on “disease–syndrome therapy formula” and it is expected to provide an effective solution to address the limitations of ischemic stroke treatment.

### Supplementary Information

Supplementary Information 1.

Supplementary Information 2.

Supplementary Information 3.

Supplementary Information 4.

Supplementary Information 5.

Supplementary Information 6.

Supplementary Information 7.

## Data Availability

The data in this study are available from the corresponding author upon reasonable request.
